# Self-medication and medication storage practices among Lira University students in Lira city, Northern Uganda

**DOI:** 10.3389/fpubh.2023.1259279

**Published:** 2023-11-03

**Authors:** Asher Emmanuel Ikwara, Humphrey Atwijukiire

**Affiliations:** ^1^Child Health and Development Centre, School of Medicine, Makerere University College of Health Sciences, Kampala, Uganda; ^2^Department of Epidemiology and Biostatistics, Faculty of Public Health, Lira University, Lira, Uganda; ^3^Department of Nursing, Faculty of Nursing and Health Sciences, Bishop Stuart University, Mbarara, Uganda

**Keywords:** antibiotic resistance, drug misuse, self-medication, storage, practices

## Abstract

**Background:**

Self-medication (SM) is a global public health concern, particularly prevalent in developing nations. It involves the consumption of drugs without professional guidance, encompassing both over-the-counter and prescription medicines. Responsible SM can alleviate healthcare burdens, reduce costs, and empower individuals to manage minor ailments independently. However, improper SM practices can lead to adverse effects, contribute to antimicrobial resistance, and pose various health risks. This study aimed to evaluate the prevalence of SM and related practices of storing medications among students at Lira University, located in Lira City West Division, Northern Uganda.

**Methods:**

This study adopted a quantitative cross-sectional design, focusing on students from various faculties. The sample size comprised 422 students, determined using the Kish Leslie formula. Data collection involved the administration of self-administered questionnaires, and subsequent data analysis was performed using SPSS version 25.

**Results:**

The study involved participants from Lira University, primarily women (51.2%) with an average age of 23.82. A majority were Christians (59.7%) and single (87.9%). Most were from the Faculty of Health Sciences (63.7%), with third-year students being the largest group (48.3%). Regarding SM, 74.2% practiced SM, with antibiotics (69.2%) and tablets (69.2%) being commonly self-administered. In terms of medication storage, 93.4% kept medicines at home, mostly in cupboards (76.5%), but many were unsure about proper storage practices. Participants exhibited good knowledge of SM's risks and measures to reduce them. There was a significant association between the faculty of study and SM practices (*p* = 0.015), while other demographic factors showed no significant associations.

**Conclusion:**

This study sheds light on the prevalent SM practices among students at Lira University in Northern Uganda. Notably, antibiotics were frequently self-medicated, raising concerns about antibiotic resistance. Additionally, medication storage practices emphasize the need for proper education on storage guidelines. However, the participants exhibited good knowledge of the risks associated with SM, suggesting the potential for effective awareness campaigns. The study recommends targeted health education programs, stricter regulations on medication sales, improved healthcare access, proper medication disposal, further research on the factors driving SM, collaborative efforts, and monitoring of antibiotic use to address this public health issue effectively.

## Introduction

Self-medication (SM) is a global public health concern (1), particularly prevalent in developing nations. It is the consumption of drugs without professional guidance, encompassing diagnosis, treatment, and supervision, commonly involving both over-the-counter (OTC) and prescription-only medicines. It also includes reusing previous prescriptions, relying on recommendations from relatives, and using leftover medications at home (1). When used effectively, SM can help alleviate the strain on healthcare services, decrease the time patients have to wait to consult a doctor, and lead to cost savings, particularly in economies with limited resources (2). The World Health Organization (WHO) underscores that responsible SM can effectively address ailments without necessitating medical consultation and offer a cost-effective alternative for common illnesses (3). Nonetheless, improper SM practices can lead to adverse drug reactions, inappropriate medication choices, the risk of double dosing or harmful interactions, potential dependence and abuse, over- or under-dosing of drugs, and the development of antimicrobial resistance due to irrational antibiotic use (4). SM manifests in various forms, such as taking medications without a physician's prescription, using a past prescription for a similar ailment, or utilizing available drugs at home without professional advice (5). Antibiotics, in particular, are frequently self-administered globally, with more than 50% obtained and used without a prescription (6).

Numerous investigations across various countries have probed SM behaviors among university students. High SM prevalence has been documented among university students in Bangladesh (88%) (7), Jordan (86.7%), Saudi Arabia (86%) (8), and Egypt (52.7%) (9). Likewise, studies among students at Gadjah Mada University, Indonesia and Jiangsu University, China unveiled that antibiotic-based SM was practiced by 49 and 47.9% of participants, respectively (10). In Ethiopia, SM prevalence among university students ranged from 32.7 to 70.8% (9).

Reasons for engaging in SM frequently cited across prior studies include past experience, cost and time savings, managing minor illnesses, seeking rapid relief, input from relatives/friends, personal convenience, easy drug availability, dissatisfaction with provided services, and discourteous healthcare professionals (5). The proliferation of pharmaceuticals worldwide has facilitated consumer access and inadvertently created opportunities for misuse (11). Rampant pharmaceutical advertising poses a significant risk of SM, especially among the younger population (12). Unregulated sales of both OTC and prescription medications perpetuate SM practices among the general populace (13).

Common irrational medication practices at home, prevalent in developing countries, encompass poor medication storage, using drugs from multiple prescribers, reusing discontinued medications, and retaining expired or no longer needed drugs. These issues stem from patients' inadequate knowledge about proper storage conditions, appropriate disposal of unused medications, and the risks linked to improper/irrational drug use (14), exerting a direct impact on public health, the environment, and healthcare systems (15). Despite the common practice of storing medications for various purposes in households worldwide, limited awareness exists regarding safe storage practices, frequently leading to irrational use (16).

The repercussions of SM are particularly pronounced in developing countries with constrained resources, low literacy rates, limited healthcare infrastructure, and a substantial segment of the population lacking access to information or sufficient therapeutic knowledge regarding dosage, duration, and potential side effects (13). SM and associated behaviors can result in various adverse effects, including intoxication, bacterial resistance, drug dependence, obscuring serious illness symptoms (17), and elevating the risks of illegal drug use and dependence, all compromising human safety (15). Youth, in particular, are vulnerable to SM (18). SM patterns are influenced by diverse factors, such as age, gender, income, self-care orientation, education level, medical knowledge, satisfaction, and perception of illness seriousness (19). Alarming rates of SM practice have been observed among younger generations, particularly college students (3). Despite regulations in some developed countries (20), global drug resistance, especially due to the misuse of antibiotics without prescriptions, remains a pressing concern (21).

While over-the-counter (OTC) drugs are designed for SM and are proven effective and safe, their improper use due to a lack of awareness about side effects and interactions can lead to severe complications (15). The prevalence of SM among students is evidently on the rise (22); for instance, a study at Mbarara University in Uganda reported a 63.5% SM prevalence (23). Although previous research on SM in Uganda has primarily focused on healthcare students, there is a dearth of information concerning the general student population. Thus, this study aims to assess SM practices among healthcare students while concurrently exploring the underlying reasons driving such practices.

## Methods and materials

### Study design

The study employed a descriptive cross-sectional design and utilized quantitative data collection and analysis techniques. The selection of a quantitative approach allowed for the exploration of various variables.

### Study site and settings

The research took place at Lira University, situated in Ayere Ward, Lira Division, within Lira city. The study was conducted between April 2022 and September 2022. Lira University covers ~500 acres of land and is situated ~11 km northwest of Lira city center on the Lira Kamudini road. The university enrolls a total of 1,378 students, comprising 662 men and 716 women. These students are distributed across three active faculties: Health Science (421 students; 196 men and 225 women), Education (219 students; 113 men and 106 women), and Management Sciences (610 students; 353 men and 257 women).

### Study procedure

Approval for the research proposal was obtained from the Gulu University Research and Ethics Committee (GUREC). Permission was sought from the Academic Registrar and the respective faculty heads before conducting the study. Convenient sampling was used, and participants were approached for consent to participate. Informed consent was obtained from all participants prior to data collection. Trained research assistants were responsible for data collection.

### Study population

The study targeted both male and female students enrolled at Lira University during the study period. Participants from all three active faculties—Health Science, Education, and Management Sciences—were included.

### Eligibility criteria (inclusion and exclusion)

The study included all registered Lira University students aged 18 years and older who consented to participate. Both male and female students were eligible, while those who were critically ill were excluded.

### Sample size determination

The study used the Kish Leslie formula from 1965 for single proportions to determine the sample size: *n* = (*Z*^2^*p(1-*p*))/*d*^2^ + non-response rate (increased by 10%), where: *n* = sample size, *Z* = 1.96 (for 95% confidence interval), *p* = 0.5 (50% proportion due to unknown current prevalence of SM), *q* = 0.05, and *d* = 0.05 (error margin); on calculation: *n* = 384.16 + (10% ^*^ 384.16) = 422.576, rounded to 422 students.

### Sampling techniques

The quantitative sample of 422 students was acquired through consecutive sampling. Trained research assistants administered questionnaires to students who consented to participate.

### Data management (data collection, cleaning, and analysis)

#### Data collection

Quantitative data were collected using self-administered questionnaires by trained research assistants. Probing was employed when needed for clarity and additional information.

#### Data entry, cleaning, and coding

The collected data were checked for completeness and entered into Excel worksheets. Post-entry data coding was done before transferring the data to SPSS version 25 for analysis.

#### Data analysis

Quantitative data, including the prevalence of SM and medication storage practices, was analyzed at the univariate level. Frequencies and proportions were used for categorical data, while continuous data like age were summarized using means and standard deviations, or medians. Bivariate analysis involved associations between SM and medication practices using chi-square tests with a 95% confidence level.

### Measurement of study variables

#### Dependent variables

SM and medication storage practices were dependent variables. SM was binary (yes/no) and assessed if participants took drugs without a prescription from a health worker within the current semester. Proper/improper medication storage was based on WHO's 2020 guidelines, including well-labeled storage, room temperature, avoiding light and particular solutions, child-proof storage, and cool and dry storage.

#### Independent variables

Independent variables included socio-demographic characteristics and behaviors toward SM and medication storage, presented as frequencies and proportions.

### Quality control (validity and reliability)

The study employed four trained research assistants for data collection, and questionnaires were pretested on 10% of the sample size. Cronbach's alpha was calculated for internal consistency. The study tools were reviewed post-data collection for completeness and errors.

### Ethical consideration

The study adhered to the Declaration of Helsinki 2013. Ethical approval was obtained from GUREC and clearance from the Uganda National Council of Science and Technology. Approval was obtained from the Academic Registrar and Faculty deans, with strict adherence to confidentiality by substituting identifiers with initials. Participants willingly gave their informed consent.

## Results

### The demographic characteristics of the study participants

According to Table 1, the average age of the participants was 23.82 ± 2.89 years. Most of the participants were women [216 (51.2%)] and 252 (59.7%) of the participants were Christians. The majority of them [371 (87.9%)] were single. Most of the participants [269 (63.7%)] were from the faculty of health sciences, and year 3 had the highest number of participants [204 (48.3%)].

**Table 1:** The characteristics of the study participants.

**Variable**	**Category**	**Frequency (*n*)**	**Percentage (%)**
Age (years)	23.82 ± 2.89		
Sex	Male	206	48.8
	Female	216	51.2
Religion	Others	120	28.4
	Christian	252	59.7
	Muslim	50	11.8
Marital status	Single	371	87.9
	Married	51	12.1
Faculty	Health sciences	269	63.7
	Education	70	16.6
	Management sciences	83	19.7
Year of study	One	56	13.3
	Two	162	38.4
	Three	204	48.3

### Participants' self-medication practices

According to Table 2, the majority of the participants [313 (74.2%)] practiced SM. Of these participants, 134 (31.8%) had practiced SM three times. The most commonly self-medicated drugs were antibiotics [292 (69.2%)], and tablets [292 (69.2%)] were the most commonly self-medicated drug formulations.

**Table 2:** Self-medication practices of the participants.

**Variables**	**Category**	**Frequency (*n*)**	**Percentage (%)**
Practiced self-medication	Yes	313	74.2
	No	109	25.8
How often have you engaged in self-medication over the past 3 months?	1	16	3.7
	2	105	24.9
	3	134	31.8
	4 and more	58	13.7
Common self-medicated drugs	Antibiotics	292	69.2
	Herbal	23	5.5
	Cold tablets	5	1.2
	Contraceptives	4	0.9
Which formulation do you usually use for self-medication?	Tablets	292	69.2
	Capsules	23	5.5
	Syrups	5	1.2
	Ointments	4	0.9
	Others	1	0.2

### Medication storage practices among participants

A significant number of participants, specifically 132 (31.3%), expressed uncertainty regarding the labeling and expiration dates of their medications. Likewise, 148 (35.1%) participants were unsure if their drugs were stored at room temperature, and 158 (37.4%) were uncertain about the storage of their medications away from light. Additionally, 148 (35.1%) were uncertain if their medicines were both stored out of reach of children and in cool and dry places.

According to Table 3, the majority of the participants [394 (93.4%)] keep medications at home, with the cupboards having the most storage [323 (76.5%)]. A significant number of participants, specifically 132 (31.3%), expressed uncertainty regarding the labeling and expiration dates of their medications. Likewise, 148 (35.1%) participants were unsure if their drugs were stored at room temperature, and 158 (37.4%) were uncertain about the storage of their medications away from light. Additionally, 148 (35.1%) were uncertain if their medicines were both stored out of reach of children and in cool and dry places.

**Table 3:** Medication storage practices.

**Variable**	**Category**	**Frequency (*n*)**	**Percentage (%)**
Stored medicines at home?	Yes	394	93.4
	No	28	6.6
Medicine storage places	Cupboard	323	76.5
	Table	15	3.6
	Bags	26	6.2
	Others	58	13.7
Stored well-labeled by names, expiry date, and dosing	Strongly agreed	82	19.4
	Agreed	42	10.0
	Disagreed	42	10.0
	Strongly disagreed	30	7.1
	Not sure	132	31.3
Stored at room temperature	Strongly agreed	43	10.2
	Agreed	71	16.8
	Disagreed	53	12.6
	Strongly disagreed	11	2.6
	Not sure	148	35.1
Stored away from light	Strongly agreed	41	9.7
	Agreed	63	14.9
	Disagreed	40	9.5
	Strongly disagreed	24	5.7
	Not sure	158	37.4
Medicines are stored out of reach of children	Strongly agreed	28	6.6
	Agreed	65	15.4
	Disagreed	39	9.2
	Strongly disagreed	44	10.4
	Not sure	148	35.1
Medicines are stored in a cool and dry place	Strongly agreed	34	8.1
	Agreed	85	20.1
	Disagreed	23	5.5
	Strongly disagreed	34	8.1
	Not sure	148	35.1

### Knowledge of the participants on self-medication

According to Table 4, of the participants, 194 (46.0%) stated that the source of information was from their academic knowledge, the majority of them [387 (91.7%)] read the instructions on manufacturer's leaflets, and 402 (95.2%) were aware that the use of leftover medication is not safe. In all, 310 (73.5%) stated that SM is not part of self-care, and 391 (92.7%) stated that SM has negative effects. The primary reasons frequently cited for SM were as follows: emergency use, with 306 individuals (72.5%) indicating this; mild illness, with 298 individuals (70.6%) opting for SM in such cases; prior prescriptions, as mentioned by 286 individuals (67.8%); and the desire to save time, with 284 individuals (67.3%) choosing SM for this reason.

**Table 4:** Knowledge toward self-medication.

**Variable**	**Category**	**Frequency (*n*)**	**Percentage (%)**
Source of information about drugs	Health work	28	6.6
	Old prescriptions	109	25.8
	Academic knowledge	194	46.0
	Others	91	21.5
Read instructions on manufacturer's leaflets	Yes	387	91.7
	No	35	8.3
Use of leftover medicines from family and is safe	Yes	20	4.7
	No	402	95.2
SM is part of self-care	Yes	108	25.6
	No	310	73.5
SM has negative consequences	Yes	391	92.7
	No	31	7.3
Reason for SM	Emergency use	306	72.5
	Drug is less expensive	274	64.9
	To save time	284	67.3
	Illness was mild	298	70.6
	Previous prescriptions	286	67.8
	Ease of access	280	66.4
	Peer influence	133	31.5
	Stressful conditions	146	34.6
Ways on how to stop/reduce SM	Prevent the supply of medicines without prescription	388	91.9
	Create awareness on side effects	394	93.4
	Enforcing strict laws on pharmacies	372	88.2
	Making health facilities easily accessible	362	85.8

The stated measures to reduce SM were as follows: prevent the supply of medicines without prescription [388 (91.9%)], create awareness on side effects [394 (93.4%)], enforce strict laws on pharmacies [372 (88.2%)], and make health facilities easily accessible [362 (85.8%)].

### Association between the demographic characteristics of the participants and SM

Chi-square statistics were used to examine the association between the demographic characteristics of the participants and SM practice (5). There was a significant relationship at the 5% significance level between the faculty of study and SM practice (*X*^2^ = 8.423, *P* = 0.015). All the other variables were insignificant as illustrated in Table 5.

**Table 5:** The association between participants' characteristics and self-medication practices.

**Variable**	**Category**	**Self-medication practices**		***X* ^2^**	**P-value**
		**Yes**	**No**		
Sex	Male	146	60	2.283	0.131
	Female	167	49		
Religion	Others	81	39	4.734	0.094
	Christian	191	61		
	Muslim	41	9		
Marital status	Single	271	100	2.027	0.154
	Married	41	9		
Faculty	Health sciences	212	57	8.423	0.015
	Education	47	23		
	Management sciences	54	29		
Year of study	One	43	13	1.984	0.371
	Two	114	48		
	Three	156	48		

## Discussion

### The demographic characteristics of the study participants

The study assessed SM and medication storage practices among Lira University students in Lira City, Northern Uganda. The study participants were 422 Lira University students from different faculties, which made it relative to the other studies cited as they had done similar work among students (22, 24). The average age of the participants was 23.82 ± 2.89 years. Most of the participants were women [216 (51.2%)], which was similar to most cited articles except for the study by Tabassum et al. (25). In all, 133 (31.5%) of the participants were Christians. This could have been a result of Lira University being based in Uganda, a country where most people are Christians. The majority of them [371 (87.9%)] were single, which was similar to other studies. Most of the participants [269 (63.7%)] were from the faculty of health sciences, and both years 2 and 3 had the highest number of participants [163 (38.6%)]. This was due to class-based knowledge, which they attributed to pharmacology lectures, which are normally taught to years 2 and 3 students at Lira University. The study participants were both men and women as in several other studies we encountered (26). Both health science students and non-health science students were employed/engaged in the study as in several other studies.

### Self-medication practices of the participants

SM is common all over the world (26), and is practiced in most countries, especially by elite groups. Our study finds that the majority of the participants [313 (74.2%)] practiced SM. Almost similar findings with varying prevalence were reported in previous studies: 63.5% was reported in a study at Mbarara University in the south-western region of Uganda (23); 64.98% in Ethiopia (27); 78.6% in India, with a larger number of women self-medicating (81.2%) than men (75.3%) (12); 71.7% in Malaysia (28); 62.9% in Egypt (1); and 98.2%, the highest prevalence reported in Saudi Arabia (29). The variations in the prevalence may be due to differences in educational background and socio-economic characteristics of study participants, clinical exposure, and variations in recall periods used. Generally, the prevalence of SM is high in most countries, and the health and pharmaceutical regulating bodies should consider measures to regulate SM.

According to our study, the most self-medicated drugs were the antibiotics [292 (69.2%)]. Similar findings were reported, showing that antibiotics (53.6%) were the most self-medicated because they were the most recommended by physicians and were frequently reused (30). Contrary to our findings, the most commonly used drugs were antipyretics and analgesics (28), which were the most common therapeutic categories for SM. In some other settings, antibiotics (31) and antiemetics were the least commonly used drugs for self-medication (31). The difference in the choice of medication to use is largely determined by the common illnesses since different geographical regions face different medical conditions.

### Medication storage practices among participants

The majority of the participants [394 (93.4%)] keep medications at home, as was similarly disclosed by a study aimed at assessing the prevalence and determinants of household medication storage during the COVID-19 outbreak in southwestern Ethiopia (14), where medicines were stored at home for future use. Similar studies in Northern Uganda (32), Ethiopia (21), and China (16) have portrayed a significant percentage of drugs being stored at home for ongoing use. In most cases, medicines were stored in cupboards. Similarly, a study in Saudi Arabia indicated that medications are widely stored in people's homes throughout the world, which is most likely to affect the effectiveness and stability of these medications (33). Antibiotics, besides being mostly self-medicated by the respondents, also turned out to be the most stored, probably indicating poor medicine adherence, which could evolve into a risk factor for antibiotic resistance (28) and treatment failure (34).

### Knowledge of the participants on self-medication

In all, 46% of our participants stated that the source of information was obtained from their academic knowledge. They obtained information through reading material, and the reasons quoted were minor ailments and quick relief. The majority of students agreed that medical knowledge is necessary for the administration of medicine by themselves. SM is highly prevalent among medical students, which is quite alarming (28). Furthermore, the majority [387 (91.7%)] of our study participants read the instructions on the manufacturers' leaflets as the source of knowledge, which is different from other reported findings, which show that the majority of people did not read the packaging inserts (30). This could be due to the characteristics of the study participants, with our study being carried out entirely among university students.

Our findings indicated that 402 (95.2%) were aware that the use of leftover medication is not safe, and 73.5% stated that SM is not part of self-care, which is higher than the findings of the study in India, which showed that only 47% of the participants opined that SM was a part of self-care (12). Furthermore, our findings showed that 391 (92.7%) stated that SM has negative effects, which is similar to the finding of another study done among students that showed that the majority (94.3%) of students knew that all medications (prescription, OTC, and herbal) had adverse effects (5). This is highly reported because the study was done among university students and, more specifically, health professional students.

The most stated reasons for SM were emergency use [306 (72.5%)], mild illness [298 (70.6%)], previous prescriptions [286 (67.8%)], and to save time [284 (67.3%)]. Similar findings were reported in various studies. The majority of the students self-medicated because the illness was too trivial for consultation (70.5%) (12). Students believed that SM can be practiced when the illness is not too serious (9). SM was related to their knowledge of their ailment and its treatment, and others thought it saved time (11). Some adolescents took SM during emergency (31) and when needing rapid relief (20) or having old prescriptions (23), and in Ethiopia, mildness of disease and dissatisfaction with health-care services were the main reasons for SM (4). Across different studies, the reasons for SMs are minor and can be solved by creating awareness on the dangers of SM and extending adolescent-friendly health services closer to students to prevent the side effects of SM.

The stated measures to reduce SM were as follows: creating awareness on side effects [394 (93.4%)], enforcing strict laws on pharmacies [372 (88.2%)], and making health facilities easily accessible [362 (85.8%)]. Other studies highlighted the impact of health education on the awareness of drugs' side effects among self-medicating students (31), and this could be a reliable measure to reduce SM.

## Conclusion

The study conducted among Lira University students in Northern Uganda revealed significant insights into SM and medication storage practices. It is evident that SM is prevalent among these students, with a majority engaging in SM, primarily driven by reasons such as the perception of minor illnesses and the need for quick relief. Antibiotics emerged as the most commonly self-medicated drugs, raising concerns about antibiotic resistance and proper medication adherence. Medication storage practices were also noteworthy, with the majority of participants keeping medications at home, often in cupboards. Proper medication storage and adherence are critical for ensuring treatment effectiveness and patient safety. The participants demonstrated a good level of knowledge regarding the risks associated with SM, and many acknowledged that SM is not a part of self-care. This indicates the potential for raising awareness about responsible medication practices.

## Recommendations

1. We recommend the implementation of comprehensive health education programs within universities to raise awareness among students about the risks and consequences of SM. These programs should emphasize the importance of consulting healthcare professionals for appropriate medical advice.
2. Enforcing strict regulations on the sale of over-the-counter (OTC) and prescription medications to prevent easy access to medications without proper medical guidance is also recommended. This includes preventing the supply of medicines without a prescription.
3. Improved accessibility of healthcare facilities and services to ensure that students have easy access to professional medical advice and treatment is among our major recommendations. This can help reduce the reliance on SM for minor illnesses.
4. We also recommend the promotion of proper disposal of unused or expired medications to discourage the use of leftover medication, which can be unsafe and contribute to SM practices. The research team also recommended conducting further research to understand the specific factors driving SM among university students, considering cultural and regional variations. This will enable the development of targeted interventions.
5. We encouraged collaboration between universities, healthcare institutions, and regulatory bodies to address SM practices comprehensively. This can involve creating policies and guidelines tailored to the university environment.
6. Given the high prevalence of antibiotic SM, implementing monitoring systems for antibiotic dispensing and usage to curb antibiotic resistance is strongly recommended.

## Strengths

This study on SM practices among Lira University students in Northern Uganda exhibits several strengths, including its relevance to public health, robust methodology, large and diverse sample, detailed demographic information, thorough data analysis, and multifaceted recommendations. These strengths enhance the study's credibility and its potential to contribute to effective interventions addressing SM in the university setting.

## Limitations

One of the researchers and the study participants were from the same university. This could have created some bias, especially among friendly participants.

## Data availability statement

The raw data supporting the conclusions of this article will be made available by the authors, without undue reservation.

## Ethics statement

The studies involving humans were approved by Gulu University Research Ethics Committee. The studies were conducted in accordance with the local legislation and institutional requirements. The participants provided their written informed consent to participate in this study. The animal study was approved by Gulu University Research Ethics Committee. The study was conducted in accordance with the local legislation and institutional requirements.

## Author contributions

AI: Conceptualization, Data curation, Formal analysis, Investigation, Methodology, Project administration, Software, Supervision, Visualization, Writing original draft, Writing review & editing. HA: Data curation, Formal analysis, Investigation, Methodology, Software, Validation, Visualization, Writing original draft.
